# Trends in prescription of new antiseizure medications in a single center in Latin America: evidence of clinical practice

**DOI:** 10.3389/fneur.2025.1562079

**Published:** 2025-05-21

**Authors:** Jimena Colado-Martinez, Jimena Gonzalez-Salido, Irving Fuentes-Calvo, Betsy C. Vázquez-Cruz, Fernando Vasquez-Lopez, Pilar Robles-Lomelin, Alan M. Solis-Velázquez, Luis A. Marin-Castañeda, Mijail A. Rivas-Cruz, Diego A. Barrios-González, Eithel Valenzuela-Mendivil, Fernando Sotelo-Díaz, Gerardo Mendez-Suarez, Salvador Martínez-Medina, Daniel A. Martínez-Piña, L. Jimena Gómez-Rodríguez, Aurelio Jara-Prado, Adriana Ochoa-Morales, Jorge Guerrero-Camacho, Michele L. Breda-Yepes, M. Nahomi Herrera-Noguera, Juan C. Reséndiz-Aparicio, Mario A. Sebastián-Díaz, Iris E. Martínez-Juárez

**Affiliations:** ^1^Epilepsy Clinic and Clinical Epileptology Fellowship, National Institute of Neurology and Neurosurgery and Faculty of Medicine, UNAM, Mexico City, Mexico; ^2^Facultad Mexicana de Medicina, Universidad La Salle, Mexico City, Mexico; ^3^Neurology Residency Program, National Institute of Neurology and Neurosurgery and Faculty of Medicine, UNAM, Mexico City, Mexico; ^4^General Surgery Residency Program, General Hospital 20 La Margarita, IMSS, Puebla, Mexico; ^5^Neurosurgery Residency Program, National Institute of Neurology and Neurosurgery, Mexico City, Mexico; ^6^Internal Medicine Residency Program, Medica Sur, Mexico City, Mexico; ^7^Department of Neurogenetics, National Institute of Neurology and Neurosurgery, Mexico City, Mexico; ^8^Priority Epilepsy Program, National Institute of Neurology and Neurosurgery, Mexico City, Mexico; ^9^Department of Nephrology, South Central High Specialty Hospital PEMEX, Mexico City, Mexico; ^10^Clinical Neurophysiology and Cognition Laboratory, National Institute of Neurology and Neurosurgery, Mexico City, Mexico; ^11^Grupo Neurológico, Neuroquirúrgico y de Columna, Hospital Ángeles Acoxpa, Mexico City., Mexico

**Keywords:** antiseizure medications, prescription, epilepsy, epileptic syndromes, LATAM

## Abstract

**Background:**

Epilepsy affects approximately 70 million people globally, with a prevalence in Mexico of 10.8 to 20 cases per thousand. Antiseizure Medications (ASM) are the first line of treatment for people with epilepsy (PWE), aiming to achieve early seizure control while minimizing adverse effects that could impact quality of life.

**Materials and methods:**

This retrospective cohort study analyzed data from 2020 to 2024 collected from medical records, clinical histories, and electronic systems, using REDCAP^®^ and SPSSV21^®^. It included all epilepsy patients treated at the National Institute of Neurology and Neurosurgery “MVS” in Mexico City. Descriptive statistics were reported as means ± standard deviations for quantitative variables and percentages for categorical variables. Bivariate analysis used the Q Cochran test for dichotomous variables and the chi-square or Fisher’s exact test for qualitative variables.

**Results:**

Of 1,192 prescriptions, third-generation ASMs accounted for the majority (53.7%), led by levetiracetam (24.1%), lamotrigine (14%), and lacosamide (6%). Second-generation ASMs comprised 42.4%, including valproate (21.5%), carbamazepine (11.3%), and clonazepam (5.5%). First-generation ASMs were less frequently prescribed (3.9%), primarily phenytoin (2.3%), primidone (1.0%), and phenobarbital (0.3%). Third-generation ASMs were the most prescribed for focal seizures (38.6%), generalized seizures (13.3%), and seizures of unknown (1.9%) or unclassified types (2.1%).

**Discussion:**

Compared to a 2012 study in the same population, which showed second-generation ASM as dominant, this study highlights a significant shift toward third-generation ASM, now representing over half of prescriptions. While valproate and carbamazepine remain versatile second-generation options, newer ASMs, such as levetiracetam, are increasingly favored.

**Conclusion:**

These findings demonstrate a preference for second- and third-generation ASMs in tertiary hospitals in Latin America, which is concordant with global trends. First-generation ASMs are still prescribed but at lower rates. These results provide insights into changing prescription practices and access to newer medications, informing future research and hospital policies.

## Highlights

- At present, the NINNMVS prescribes third (53.7%) and second-generation (42.4%) ASM principally.
- Levetiracetam, Valproate, and Lamotrigine account for the most prescribed ASM in PWE at our center.
- Despite the availability of newer medications, first-generation ASMs remain in clinical use, accounting for 3.9% of prescriptions.
- These trends in the prescription of new-generation ASMs in LATAM align with their worldwide use in PWE.

## Introduction

Epilepsy is a common neurological disorder; it affects around 70 million people worldwide and is a common cause of disability and increased healthcare costs (1). Latin America (LATAM) is not the exception; around 6·3 million people in this continent have active epilepsy (2). Specifically in Mexico, there is a prevalence of epilepsy of over 10.8 to 20 cases per thousand people (3). The main objective of epilepsy treatment is to have early seizure control by starting medication as soon as the patient has been diagnosed while avoiding adverse effects that could diminish the quality of life. The first line of treatment for people with epilepsy (PWE) consists of antiseizure medications (ASM) worldwide; there are 25 ASM available (4). Treatment selection depends on the individual characteristics of the patient, such as age, sex, the desire to get pregnant, comorbidities, and tolerability, as well as the characteristics of the disease, such as seizure type or the diagnosis of an epileptic syndrome (1).

Until 1990, there were only six ASMs in existence, which are now called “First Generation” ASMs and include, in alphabetical order: benzodiazepines (BZD), carbamazepine (CBZ), phenobarbital (PB), phenytoin (PTH), primidone (PRM), and valproate (VPA). Throughout the years, new medications have been introduced in the market, “Second Generation” ASM include: Lamotrigine (LTG), Levetiracetam (LEV), Oxcarbazepine (OXC), and Topiramate (TPM) (4). And newer “Third Generation” ASMs are now available: Brivaracetam (BRV), Eslicarbazepine (ESL), and Lacosamide (LCS) (4). The use of first-generation ASMs, such as CBZ or VPA, as the first line of treatment in PWE is still a practice worldwide. However, the availability of new ASMs has led physicians to expand their treatment options (5). Although the SANAD II study has shown similar efficacies between first—and second-generation ASM, the later ones have fewer adverse effects, which can improve treatment adherence and patient’s quality of life (6).

The Epilepsy Priority Program (EPP) in Mexico established a Multicenter Epilepsy Registry from March 2021 to December 2022, it consisted of 6,653 patients all over the country. The Epilepsy Clinic, along with the Clinical Epileptology Fellowship at NINNMVS, participated in the project and facilitated the recollection of sociodemographic and clinical data from PWE (7).

The following study described the use of new-generation ASM in the subgroup of PWE in the EPP Multicenter Epilepsy Registry who attended the Epilepsy Clinic at NINNMVS in Mexico City and contrasts it with the use of previous ASM in the same population. In Mexico, health access is divided into three groups. Government health insurance is available for Government employees through the Institute for Social Security and Services for State Workers (ISSSTE), general employees through the Mexican Institute of Social Security (IMSS), and the uninsured population through the Ministry of Health (SSA - *Secretaría de Salud*). The rest of the population pays for health insurance through private companies. (8) Private health insurance companies have access to all ASMs, including newer generations (except cannabidiol and cenobamate, which are not available in the country). Government-run health institutes have a more limited availability, especially in primary care centers, where available ASMs are included annually in a “basic catalog of medication.” In 2024, for example, it included VPA, LEV, PHT, PB, CBZ, TPM, and OXC. Newer generation ASMs are only available in the tertiary care level, such as the NINNMVS, which includes BVC, BZD, LTG and LCM (9).

## Materials and methods

### Study design

This study aimed to determine the use of new-generation ASM in a referral hospital in LATAM. An observational retrospective cohort from 2021 through 2024 was obtained from the EPP Multicenter Epilepsy Registry. The study had the approval of NINN Bioethics and Research Committees No. 68/21.

### Patients

The study included PWE, a subgroup of the EPP registry, who were evaluated by one Epileptologist (I.E.M.J.) and Clinical Epileptology Fellows in the Epilepsy Clinic at the NINNMVS. Data were collected from patients’ medical records up to their most recent follow-up and entered into REDCap^®^ for organization and storage. The data were then analyzed using SPSS^®^ version 21. Clinical and sociodemographic data of PWE were obtained. Seizure types were classified according to the 2017 ILAE classification system (10).

### Use of antiseizure medications

**Figure 1:**
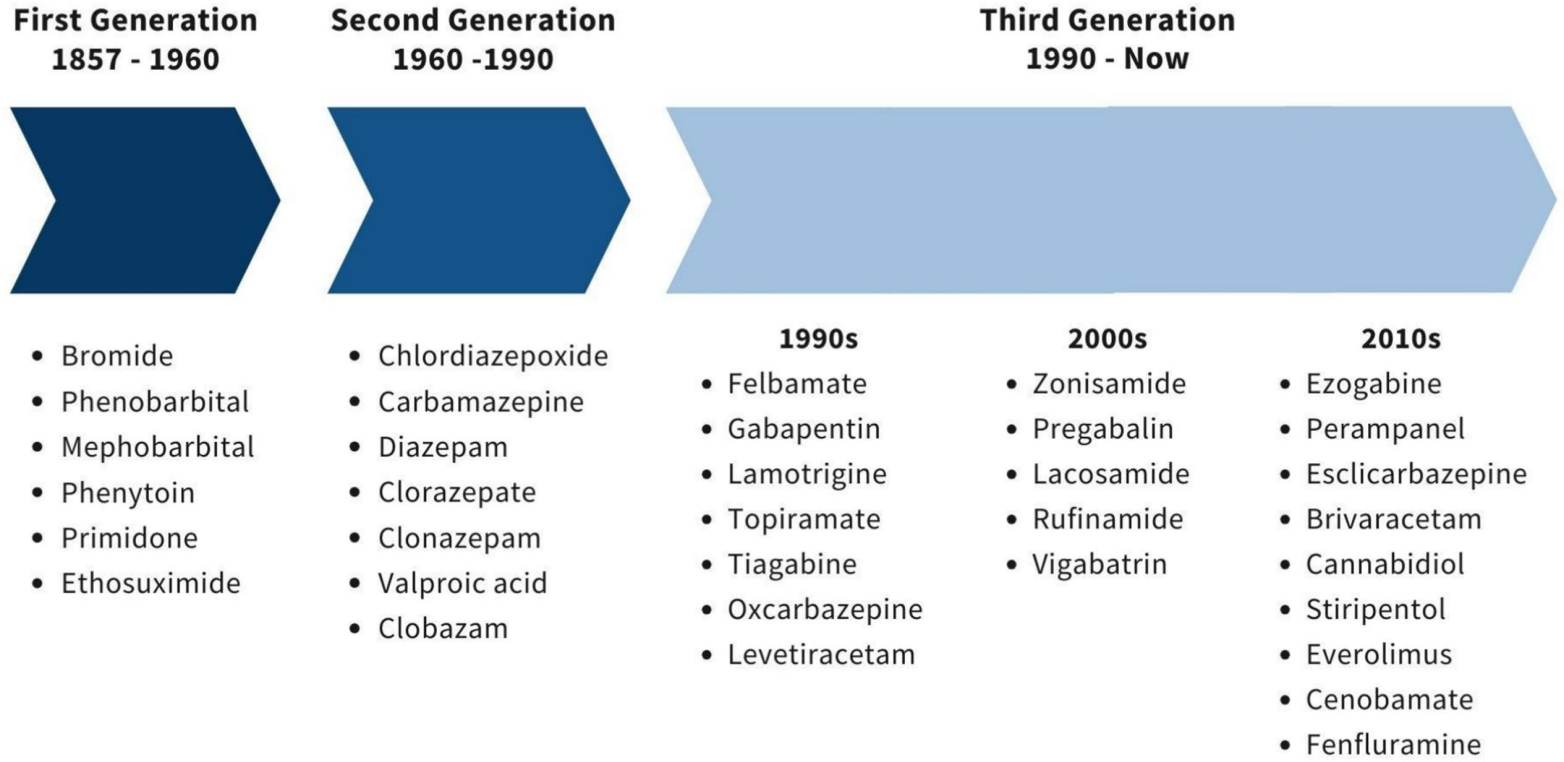
Classification of ASM generation according to Gunasekera. ASM, Anti-seizure medications. Figure modified from Gunasekera’s classification (22).

Current and previous ASM use in PWE was analyzed. Previous ASMs refer to those prescribed by other institutions or physicians before the patient’s first visit to our clinic, based on their prior treatment regimen. Antiseizure medications were divided into three classes: (I) First generation, (II) Second generation, and (III) Third generation, based on Gunasekera’s classification (9) (see Figure 1).

### Sample and statistical analysis

Descriptive analysis was performed for quantitative variables with means and standard deviation, while percentages and proportions were used for qualitative variables. Bivariate analysis was conducted using the Q Cochran test for dichotomous dependent variables and either the Chi-square test or Fisher’s exact test for qualitative variables, and Student’s t test or its equivalent non-parametric were used for quantitative variables, all analysis was done using SPSS^®^ version 21.

## Results

### Sociodemographic and clinical characteristics

A group of 635 patients from the EPP Multicenter Epilepsy Registry who attended the NINNMVS Epilepsy Clinic were included. Among them, 378 were female (59.5%), and 257 were male (40.5%). The patients’ mean age was 36.88 ± 13.4 (15–79). The mean time since epilepsy diagnosis to inclusion in the registry was 12.95 ± 6.9 years (6 months - 25 years). The majority of patients were on monotherapy (253, 39.8%), while 271 (42.7%) were prescribed multiple ASMs. The sociodemographic and clinical characteristics of the PWE, including education level, past medical history, seizure type, epilepsy syndrome and or epilepsy etiologies, and epilepsy surgery are summarized in Table 1.

**Table 1:** Characteristics of patients with epilepsy included in this study.

Patients characteristics *n* = 635		
Sociodemographic data		
		n (%)
Sex	Female	378 (59.5%)
	Male	257 (40.5%)
Age	In years (mean ± SD)	36.88 ± 13.4
Highest education level	None	62 (9.8%)
	Elementary school	87 (13.7%)
	Secondary school	143 (22.5%)
	High school	163 (25.7%)
	University	122 (19.2%)
	Postgraduate degree	11 (1.7%)
	Special education	21 (3.3%)
Clinical data		
Time with the diagnosis of epilepsy	Years (mean ± SD)	12.95 ± 6.9
History	Febrile seizures	58 (9.1%)
	Family history of epilepsy	84 (13.2%)
Seizure type	Focal onset	429 (67.6%)
	Generalized onset	174 (27.4%)
	Unknown onset	29 (4.6%)
	Unclassified seizures	3 (0.5%)
Etiology	Structural	368 (58%)
	Genetic	98 (15.4%)
	Infectious	13 (2%)
	Metabolic	2 (0.3%)
	Inmune	25 (3.9%)
	Unknown	161 (25.4%)
Epilepsy surgery	Corpus Callosotomy	8 (1.3%)
	Vagal Nerve Stimulator (VNS)	1 (0.2%)
	Hemispherectomy	5 (0.8%)
	Lesionectomy	21 (3.3%)
	Temporal lobectomy	38 (6%)
	Extra Temporal Resection	3 (0.5%)
	Transsphenoidal Resection	8 (1.3%)
Electroencephalogram	Total available	410 (54.6%)
	Normal	166 (26.1%)
	Abnormal	244 (38.44%)
Seizure frequency	Seizure free	249 (39.2%)
	1–3 seizures per month	310 (48.8%)
	4–6 seizures per month	50 (7.9%)
	>10 seizures per month	26 (4.1%)
Number of ASM	Monotherapy; n (%)	253 (39.8%)
	Polytherapy	271 (42.7%)
	2 ASMs	160 (25.2%)
	3 ASMs	83 (13.1%)
	4 ASMs	23 (3.6%)
	5 ASMs	5 (0.8%)

### Current use of antiseizure medications

**Figure 2:**
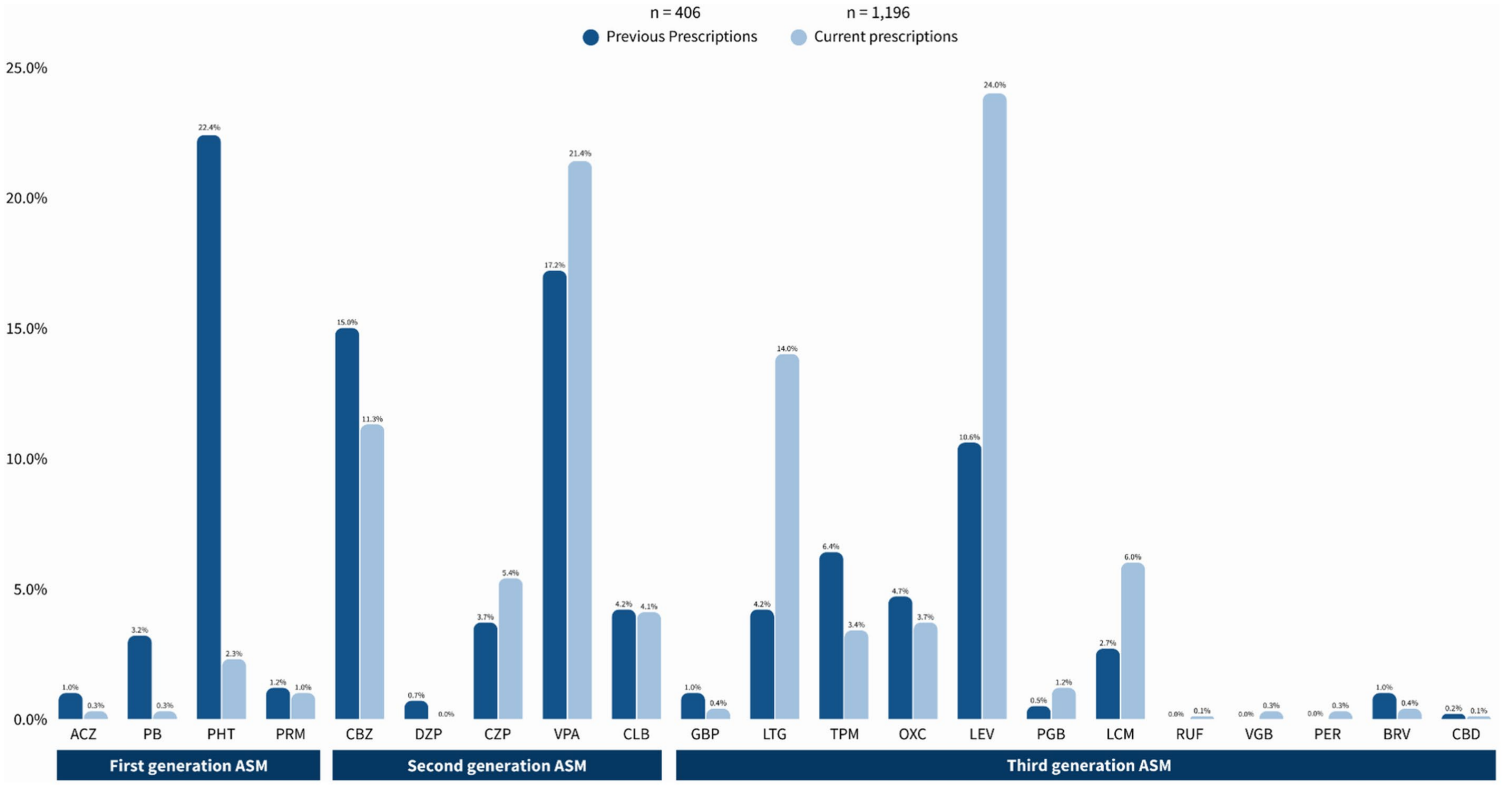
Current trends in prescriptions of anti-seizure medications drugs at NINNMVS Epilepsy Clinic. ASM, Anti-seizure medication.

A total of 1,192 prescriptions were provided at the NINNMVS Epilepsy Clinic throughout the study. Of these, 47 (3.9%) prescriptions were for first-generation ASM, with PHT being the most commonly prescribed (28, 2.3%), followed by PRM (12, 1.0%) and PB (4, 0.3%). Meanwhile, 505 prescriptions (42.4%) were for second-generation ASM, primarily VPA (256, 21.5%), CBZ (135, 11.3%), and CZP (65, 5.5%). Finally, third-generation ASM accounted for 640 prescriptions (53.7%), including LEV (287, 24.1%), LTG (167, 14%), and LCM (72, 6%) (Figure 2).

### Previous use of antiseizure medications

Table 2 compares previous ASM use before attending our center with current use, where applicable, in the same population of PWE. Of a total of 406 prescriptions, 113 (27.8%) were First-Generation ASM, primarily PHT (91, 22.4%) and PB (12, 3.2%). Second-generation ASMs accounted for 166 (40.9%) of the prescriptions, with VPA (70, 17.2%) and CBZ (61, 15%) being the most common. Finally, Third-Generation ASM constituted 127 (31.3%) of the total prescriptions, with the most prevalent being LEV (43, 10.6%), LTG (17, 4.2%), and LCM (11, 2.7%).

**Table 2:** Previous and current use of antiseizure medications in PWE attended at NINNMVS.

Prescription of antiseizure medications					
Class of ASM (International abbreviation)			Previous prescriptions of ASM *n* = 406 n (%)	Current prescriptions of ASM *n* = 1,196 n (%)	*p*
First Generation		Acetazolamide (ACZ)	4 (1.0%)	3 (0.3%)	0.705
		Phenobarbital (PB)	13 (3.2%)	4 (0.3%)	0.020
		Phenytoin (PHT)	91 (22.4%)	28 (2.3%)	<0.001
		Primidone (PRM)	5 (1.2%)	12 (1.0%)	0.071
Second generation		Carbamazepine (CBZ)	61 (15%)	135 (11.3%)	<0.001
		Diazepam (DZP)	3 (0.7%)	0 (0%)	-
		Clonazepam (CZP)	15 (3.7%)	65 (5.4%)	<0.001
		Valproate (VPA)	70 (17.2%)	256 (21.4%)	<0.001
		Clobazam (CLB)	17 (4.2%)	49 (4.1%)	
Third generation	1990	Gabapentin (GBP)	4 (1.0%)	5 (0.4%)	0.705
		Lamotrigine (LTG)	17 (4.2%)	167 (14.0%)	<0.001
		Topiramate (TPM)	26 (6.4%)	41 (3.4%)	0.063
		Oxcarbazepine (OXC)	19 (4.7%)	44 (3.7%)	0.001
		Levetiracetam (LEV)	43 (10.6%)	287 (24.0%)	<0.001
	2000	Pregabalin (PGB)	2 (0.5%)	14 (1.2%)	0.003
		Lacosamide (LCM)	11 (2.7%)	72 (6.0%)	<0.001
		Rufinamide (RUF)	0 (0%)	1 (0.1%)	-
		Vigabatrin (VGB)	0 (0%)	3 (0.3%)	-
	2010	Perampanel (PER)	0 (0%)	4 (0.3%)	-
		Brivaracetam (BRV)	4 (1.0%)	5 (0.4%)	0.739
		Cannabidiol (CBD)	1 (0.2%)	1 (0.1%)	1.000

As for first-generation ASM, a significant reduction was observed in the prescription of PB (13 vs. 4, *p* = 0.020) and PHT (91 vs. 18, *p* < 0.001), while others, such as ACZ and ESM, did not show statistically significant differences. In second-generation ASM, there was a notable increase in the use of CBZ (61 vs. 135, *p* < 0.001), CZP (15 vs. 65, *p* < 0.001), VPA (70 vs. 256, *p* < 0.001), and CLB (17 vs. 49, *p* < 0.001). Meanwhile, for third-generation ASM, drugs such as LTG (17 vs. 167, *p* < 0.001) and LEV (43 vs. 287, *p* < 0.001) showed significant increases, whereas TPM presented a non-significant increase (26 vs. 41, *p* = 0.063).

Of the 635 patients, 379 (59.7%) had not received any treatment before their assessment at the Epilepsy Clinic. During follow-up in the NINNMVS, only 22 patients (3.5%) remained untreated due to the patient’s desire not to take ASM or due to non-compliance with treatment.

Table 3 shows a comparison between the prescription of different generations of ASM by seizure type. For focal seizures, third-generation ASMs were the most prescribed, with 462 (38.6%) prescriptions, primarily LEV (192, 16.1%) and LTG (124, 10.4%). This was followed by second-generation ASM with 355 (29.7%) prescriptions, mainly VPA (151, 12.6%) and CBZ (116, 9.7%). First-generation ASMs were the least prescribed, with 34 (3%) prescriptions, namely PHT (21, 1.8%). For generalized seizures, third-generation ASM also dominated, with 157 (13.3%) prescriptions, primarily LEV (82, 6.9%), followed by LTG (36, 3%) and TPM (13, 1.1%). Second-generation ASM followed with 135 (11.2%) prescriptions, mainly VPA (96, 8%) and CBZ (16, 1.3%). First-generation ASMs accounted for only 11 (0.9%) prescriptions, mainly for PHT (6, 0.5%) and PRM (5, 0.4%). For seizures of unknown type, third-generation ASMs were again the most prescribed, with 23 (1.9%) prescriptions, primarily LEV (11, 0.9%) and LTG (7, 0.6%). Second-generation ASM followed with 15 (1.5%) prescriptions, mainly VPA (9, 0.9%). First-generation ASMs had only 1 (0.1%) prescription. Finally, for unclassified seizures, only LEV was prescribed, with 2 (0.2%) prescriptions.

**Table 3:** Antiseizure medications prescription according to seizure type based on the 2017 ILAE seizure classification.

Antiseizure medications prescription					
*n* = 1,196 n (%)				Total n (%)	*p*
Focal seizures	First generation	Acetazolamide	3 (0.3%)	34 (3%)	0.488
		Phenobarbital	3 (0.3%)		
		Phenytoin	21 (1.8%)		
		Primidone	7 (0.6%)		
	Second generation	Carbamazepine	116 (9.7%)	355 (29.7%)	
		Clonazepam	50 (4.2%)		
		Valproate	151 (12.6%)		
		Clobazam	38 (3.2%)		
	Third generation	Gabapentin	5 (0.4%)	462 (38.6%)	
		Lamotrigine	124 (10.4%)		
		Topiramate	27 (2.3%)		
		Oxcarbazepine	36 (3.0%)		
		Levetiracetam	192 (16.1%)		
		Pregabalin	11 (0.9%)		
		Lacosamide	58 (4.8%)		
		Vigabatrin	1 (0.1%)		
		Perampanel	4 (0.3%)		
		Brivaracetam	4 (0.3%)		
Generalized	First generation	Phenytoin	6 (0.5%)	11 (0.9%)	0.539
		Primidone	5 (0.4%)		
	Second generation	Carbamazepine	16 (1.3%)	135 (11.2%)	
		Clonazepam	13 (1.1%)		
		Valproate	96 (8.0%)		
		Clobazam	10 (0.8%)		
	Third generation	Lamotrigine	36 (3.0%)	157 (13.3%)	
		Topiramate	13 (1.1%)		
		Oxcarbazepine	8 (0.7%)		
		Levetiracetam	82 (6.9%)		
		Pregabalin	3 (0.3%)		
		Lacosamide	10 (0.8%)		
		Rufinamide	1 (0.1%)		
		Vigabatrin	2 (0.2%)		
		Brivaracetam	1 (0.1%)		
		Cannabidiol	1 (0.1%)		
Unknown	First generation	Phenobarbital	1 (0.1%)	1 (0.1%)	0.830
	Second generation	Carbamazepine	3 (0.3%)	15 (1.5%)	
		Clonazepam	2 (0.2%)		
		Valproate	9 (0.9%)		
		Clobazam	1 (0.1%)		
	Third generation	Lamotrigine	7 (0.6%)	23 (1.9%)	
		Topiramate	1 (0.1%)		
		Levetiracetam	11 (0.9%)		
		Lacosamide	4 (0.3%)		
Unclassified	Third generation	Levetiracetam	2 (0.2%)	2 (0.2%)	-
No treatment		None	22 (3.5%)	22 (3.5%)	-

### Seizure freedom and use of ASMs

Table 4 summarizes de baseline seizure frequency patients presented before initiating treatment in our clinic, and seizure frequency after starting treatment. Before attending, 310 (48.8%) of the patients had between one and three seizures per month, and 249 (39.2%) were seizure-free at the time of first visit. After starting treatment in our clinic, 403 (63.5%) patients achieved seizure freedom, while 227 (35.7%) persisted with seizures.

**Table 4:** Baseline and current seizure frequency of studied patients.

Seizure frequency and use of ASMs		
n = 635 n (%)		Total n (%)
Seizure frequency before treatment in Epilepsy Clinic	Seizure free; n (%)	249 (39.2%)
	1–3 seizures per month	310 (48.8%)
	4–6 seizures per month	50 (7.9%)
	>10 seizures per month	26 (4.1%)
Seizure frequency after treatment in Epilepsy Clinic	Seizure freedom	403 (63.5%)
	Persistent seizures	227 (35.7%)
	NS	5 (0.8%)

Table 5 summarizes seizure freedom and persistence for each prescribed ASM, with percentages representing the proportion of patients within each ASM group experiencing either outcome.

**Table 5:** Seizure freedom and persistence according to prescribed ASM.

Seizure freedom/persistence and use of ASMs					
n = total of prescriptions of each ASM			n (%)	Total n (%)	*p*
Seizure freedom	First generation	ACZ: 3 prescriptions	1 (33.3%)	27 (57.4%)	**0.004**
		PB: 4 prescriptions	1 (25.0%)		
		PHT: 28 prescriptions	20 (71.4%)		
		PRM: 12 prescriptions	5 (41.6%)		
	Second generation	CBZ: 135 prescriptions	74 (54.8%)	277 (54.9%)	**<0.001**
		DZP: 0 prescriptions	-		
		CZP: 65 prescriptions	31 (47.7%)		
		VPA: 256 prescriptions	152 (59.4%)		
		CLB: 49 prescriptions	20 (40.8%)		
	Third generation	GBP: 5 prescriptions	4 (80.0%)	369 (57.3%)	0.035
		LTG: 167 prescriptions	91 (54.5%)		
		TPM: 41 prescriptions	15 (36.6%)		
		OXC: 44 prescriptions	29 (65.0%)		
		LEV: 287 prescriptions	175 (61.0%)		
		PGB: 14 prescriptions	9 (64.3%)		
		LCM: 72 prescriptions	40 (55.6%)		
		RUF: 1 prescriptions	1 (100%)		
		VGB: 3 prescriptions	1 (33.3%)		
		PER: 4 prescriptions	0 (0.0%)		
		BRV: 5 prescriptions	3 (60.0%)		
		CBD: 1 prescriptions	1 (100%)		
Persistent seizures	First generation	ACZ: 3 prescriptions	2 (66.7%)	20 (4.3%)	0.565
		PB: 4 prescriptions	3 (75.0%)		
		PHT: 28 prescriptions	8 (28.6%)		
		PRM: 12 prescriptions	7 (58.3%)		
	Second generation	CBZ: 135 prescriptions	61 (45.2%)	225 (44.6%)	**<0.001**
		DZP: 0 prescriptions	-		
		CZP: 65 prescriptions	34 (52.3%)		
		VPA: 256 prescriptions	101 (39.5%)		
		CLB: 49 prescriptions	29 (59.2%)		
	Third generation	GBP: 5 prescriptions	1 (20.0%)	271 (42.1%)	0.015
		LTG: 167 prescriptions	74 (44.3%)		
		TPM: 41 prescriptions	26 (63.4%)		
		OXC: 44 prescriptions	15 (34.1%)		
		LEV: 287 prescriptions	110 (38.3%)		
		PGB: 14 prescriptions	5 (35.7%)		
		LCM: 72 prescriptions	32 (44.4%)		
		RUF: 1 prescriptions	0 (0.0%)		
		VGB: 3 prescriptions	2 (66.7%)		
		PER: 4 prescriptions	4 (100%)		
		BRV: 5 prescriptions	2 (40.0%)		
		CBD: 1 prescriptions	0 (0.0%)		

The analysis revealed significant differences in seizure freedom based on the generation of ASMs. First-generation (*p* = 0.004) and second-generation (*p* < 0.001) drugs showed statistically significant seizure freedom rates. In terms of percentage, first- and third-generation ASMs had the highest seizure freedom rates among the prescribed medications (57.4 and 57.3%, respectively), with LEV (61.0%), VPA (59.4%), and PHT (71.4%) standing out.

Regarding seizure persistence, significant differences were found for second-generation ASMs (p < 0.001), with a higher frequency of persistent seizures in users of PB, CLB, TPM, and PER. No significant differences were observed for first- and third-generation drugs (*p* = 0.565 and *p* = 0.015, respectively).

## Discussion

### Historical prescription trends

This study represents a new update on ASM prescription trends in a real-life setting in a LATAM country in this millennium. A study conducted in Mexico at NINNMVS by Martínez-Juárez et al. in 2012 (11) examined 206 patients to evaluate the frequency of drug-resistant epilepsy and the ASM prescribed in this population. The most commonly used ASMs were second-generation drugs such as VPA and CBZ either in mono or polytherapy, with a very slight tendency towards using third-generation ASMs as adjunctive therapy.

### Study key findings

In our study, we compared patients’ previous treatment regimens before attending our clinic with those prescribed there to identify prescribing trends at our national third-level referral center. Third-generation ASMs represented 53.7% of the prescriptions, indicating their significant prevalence in clinical practice and widespread use compared to first and second-generation ASMs. Levetiracetam (LEV) and lamotrigine (LTG) demonstrated a statistically significant increase in their use in PWE attended at NINN; this may be due to their efficacy, safety profiles, and clinical acceptance. The remaining prescriptions were for second-generation ASM, suggesting a high prevalence and potential preference for these medications in the studied population. Among this group, VPA, CZP, CLB, and CBZ were the most commonly used with a statistical significance, where VPA stood out as the most versatile medication overall. Its broad-spectrum and efficacy makes it suitable for treating almost all types of seizures and epilepsy syndromes, despite its association with a risk of congenital malformations (12). This shows a trend to use newer medications; however, as they may not be as widely available or due to physician preference for medications with a longer and well-documented history of use, second-generation ASM still accounts for a wide amount of prescriptions.

Furthermore, our analysis revealed that first- and second-generation drugs were associated with statistically significant seizure freedom rates. However, when considering percentages, first- and third-generation ASMs demonstrated the highest seizure freedom rates, with LEV (61.0%), VPA (59.4%), and PHT (71.4%) standing out. In contrast, second-generation ASMs showed statistically significant rates of persistent seizures, particularly among patients prescribed PB, CLB, TPM, and PER. These outcomes may be influenced by various factors, including the different combinations of ASMs, patient adherence to treatment, drug-resistant epilepsy, and other variables that can affect the efficacy of the medications. Additionally, some medications show higher rates of both seizure freedom and persistent seizures, which could be attributed to their lower prescription frequency or their enhanced therapeutic effect when combined with other ASMs.

### Global perspectives on antiseizure medications use

To our knowledge, few studies in LATAM address the current trends in the prescription of ASM in PWE. Comparable findings were observed by Assis et al. in Brazil, where there was a significant rise in the adoption of new-generation ASMs, such as LTG and LEV, and a drop in suboptimal ASM prescriptions from 73.3 to 51.5% (13). A study in another developing country by Joshi et al. in 2020 found that VPA and CZP were preferred for generalized seizures, while CLB, CBZ, OZC, and LCM were more commonly prescribed for focal seizures in a population from India (14). In our study, third-generation ASMs, mainly LEV and LTG, were prescribed for both focal and generalized seizures. Second-generation ASMs, such as VPA and CBZ, played a significant role in the treatment of both seizure types.

In developed countries, a study by Bolin et al. (15) in Sweden revealed a significant increase in the use of third-generation ASMs; LEV was the most commonly prescribed medication for initial treatment, with its use rising from 10% in 2010 to 55% in 2022. Lamotrigine (LTG) also demonstrated strong adherence rates, with the highest number of patients remaining on their initial therapy. Meanwhile, CBZ and VPA experienced a marked decline in use, dropping from 35 to 5% and 20 to 5%, respectively.

Another study conducted in Canada by Leong et al. reported a dramatic rise in the use of newer ASMs from 0.3 to 15 per 1,000 prescriptions, with LTG playing a significant role, while older ASMs declined from 7.5 to 6.4 per 1,000 prescriptions (16). Similarly, research by Bensken and Sánchez Fernández (17) in the United States highlighted a decreased use of first-generation ASMs and a substantial rise in the prescription of LCM, similar to the findings described in this study.

Other studies corroborate these trends. For instance, Powell et al. in the UK reported a reduced use of CBZ and increased use of LEV and LTG over time (18). Jin et al. in Japan also reported a statistically significant increase in newer ASMs and a decline in the older ones, with the prescription of LEV rising from 15.6 to 22.6%. However, VPA remained the most prescribed ASM in that study (19).

In contrast, a study by Lavu et al. (20) in Canada reported only a 0.09% increase in the use of new-generation ASMs following the COVID-19 pandemic, alongside a 5.11% decrease in overall ASM prescriptions, excluding GBP and CZP. Interestingly, our study observed a significant increase in the prescription of GBP and CZP.

These findings suggest that Mexico is leaning towards global trends. Our study revealed a high prevalence and increasing use of LEV and LTG, with VPA remaining the most widely used ASM overall. Several situations could explain this trend towards the use of newer ASMs in Mexico. As explained previously, the healthcare system does not have all the ASMs available, and in primary care centers or rural areas, the use of PHT and VPA is still frequent. However, since NINNMVS is a tertiary care center, newer ASMs have become available for prescription, especially for people with drug-resistant epilepsy. The population cared for in our center corresponds to low and middle income, these people usually cannot afford private health insurance or buying new ASMs such as BVC, LTG, and LCM. Therefore, the inclusion of newer ASMs in the hospital’s medication catalog has probably enabled physicians to lean toward global prescription trends and increase the use of third-generation ASMs. However, in order to confirm this assumption, further studies should be conducted and include our patients and physicians.

### Current and future directions in the use of antiseizure medications

Epilepsy has a significant socio-sanitary impact, as it reduces the patient’s quality of life and life expectancy by 2 to 10 years and increases the mortality rate by 2 to 3 times compared to individuals without the disease. Despite the availability of newer medications, first-generation ASMs remain in clinical use, accounting for 3.9% of prescriptions, with both PB and PHT showing a significant reduction compared to previous decades.

According to these findings, and after analyzing the ASM market in the United States, it was observed that first-generation ASMs continue to be prescribed despite the increasing use of second and third-generation alternatives. This trend highlights a shift away from these first-generation medications due to safety concerns and proven effectiveness. However, their continued use may reflect limitations in accessing newer ASMs, such as economic constraints or availability issues.

The World Health Organization’s Intersectoral Global Action Plan on Epilepsy and Other Neurological Disorders emphasizes addressing these challenges to ensure quality treatment for PWE. The plan proposes strategies to reduce the treatment gap by improving access to ASMs making them more available and affordable, ensuring their safety and their high quality (21).

## Limitations

This study does not specifically address differences between brand-name and generic ASMs, nor does it distinguish between regular and extended-release formulations. Additionally, it does not emphasize seizure freedom or the duration of treatment and retention for each ASM. Instead, its primary aim is to describe current prescription trends within our clinic in a LATAM country.

## Conclusion

The study indicates that the use of second- and third-generation ASM is preferred in a tertiary hospital in LATAM, aligning with trends observed in other developing and developed countries. First-generation ASMs are still prescribed, although at a lower rate. The high prevalence of second-generation ASMs, particularly LEV and LTG, suggests a shift towards the newer medications, but first-generation ASMs, such as VPA, are still prescribed. This evidence highlights statistically significant changes in prescription trends within the same population and may inform future research and prescription policies at the hospital, as well as provide insights into the factors influencing treatment choices and access to the latest-generation medications.

## Data Availability

The original contributions presented in the study are included in the article/supplementary material, further inquiries can be directed to the corresponding author.
